# Petanin Potentiated JNK Phosphorylation to Negatively Regulate the ERK/CREB/MITF Signaling Pathway for Anti-Melanogenesis in Zebrafish

**DOI:** 10.3390/ijms25115939

**Published:** 2024-05-29

**Authors:** Jian Ouyang, Na Hu, Honglun Wang

**Affiliations:** 1 Qinghai Provincial Key Laboratory of Tibetan Medicine Research and CAS Key Laboratory of Tibetan Medicine Research, Northwest Institute of Plateau Biology, Xining 810008, China; ygzjj@126.com (J.O.); huna@nwipb.cas.cn (N.H.); 2 Huzhou China-Science Innovation Centre of Plateau Biology, Huzhou 313000, China; 3 University of Chinese Academy of Sciences, Beijing 100049, China

**Keywords:** acylated anthocyanins, tyrosinase inhibitor, anti-melanogenesis, network pharmacology, MAPKs

## Abstract

Petanin, an acylated anthocyanin from the Solanaceae family, shows potential in tyrosinase inhibitory activity and anti-melanogenic effects; however, its mechanism remains unclear. Therefore, to investigate the underlying mechanism of petanin’s anti-melanogenic effects, the enzyme activity, protein expression and mRNA transcription of melanogenic and related signaling pathways in zebrafish using network pharmacology, molecular docking and molecular dynamics simulation were combined for analysis. The results showed that petanin could inhibit tyrosinase activity and melanogenesis, change the distribution and arrangement of melanocytes and the structure of melanosomes, reduce the activities of catalase (CAT) and peroxidase (POD) and enhance the activity of glutathione reductase (GR). It also up-regulated JNK phosphorylation, inhibited ERK/RSK phosphorylation and down-regulated CREB/MITF-related protein expression and mRNA transcription. These results were consistent with the predictions provided through network pharmacology and molecular docking. Thus, petanin could inhibit the activity of tyrosinase and the expression of tyrosinase by inhibiting and negatively regulating the tyrosinase-related signaling pathway ERK/CREB/MITF through p-JNK. In conclusion, petanin is a good tyrosinase inhibitor and anti-melanin natural compound with significant market prospects in melanogenesis-related diseases and skin whitening cosmetics.

## 1. Introduction

Melanin is produced by a characteristic organelle named the melanin body within the melanocytes, located in the lower layer of keratinocytes, and it is distributed through the dendrites of melanocytes among the melanocytes and keratinocytes. It represents an important pathway for skin pigmentation in the epidermis [1,2,3]. Tyrosinase family proteins, such as tyrosinase, tyrosinase-related protein-1 (TRP-1) and TRP-2, play a critical role in the biosynthesis of melanin [4]. In particular, tyrosinase is a key rate-limiting enzyme that regulates melanin production through the promotion of the conversion of tyrosine to dihydroxyphenylalanine (DOPA), as well as further oxidation of DOPA to DOPA quinone. DOPA quinone, as a substrate for melanin synthesis, is converted by tyrosinase, TRP-1 and TRP-2 to produce melanin [5]. Meanwhile, the amount and type of melanin synthesized in the body are subject to multifactorial regulation through both internal factors, such as genetic predisposition, inflammation and hormonal balance, and external factors, such as contact with allergens or exposure to UV radiation [6,7]. However, inhibiting melanin production in melanocytes is believed to help prevent this variability and is considered to have a whitening effect. The traditional active ingredients for whitening skin care, such as kojic acid, arbutin and niacinamide, have significant active effects but also have the potential for negative side effects through their strong cytotoxicity and sensitization action [8]. With the increasing demand for safe and effective natural cosmetic raw materials, this has become a research hotspot in the development of whitening cosmetics at home and abroad.

Anthocyanins are a class of water-soluble pigments from plants, which have significant anti-melanogenesis potential [9]. *Hibiscus syriacus* L. anthocyanin extract could down-regulate the activity of mushrooming tyrosinase, significantly reduce melanin production in B16F10 cells, down-regulate the expression levels of α-MSH-induced MITF and tyrosinase and attenuate the pigmentation of zebrafish larvae. Moreover, the extract activated the phosphorylation of extracellular signal-regulated kinase (ERK), which was completely reversed with the ERK inhibitor PD98059 [10]. Cyanidin 3-*O*-galactoside was the main compound in *Pistacia vera* L. extract and effectively inhibited the activities of mushroom tyrosinase monophenolase and diphenolase (IC_50_ were 141.07 and 116.08 μg mL^−1^, respectively) and reduced the pigmentation of zebrafish embryos at the early development stage (inhibition rate was 60.01% of the control group) [11]. Cyanidin 3-*O*-rhamnoside and pelargonidin 3-*O*-rhamnoside, the main anthocyanins in the polyphenol extract from *Malpighia emarginata* DC. fruit, significantly reduced UVB-irradiated skin pigmentation, reduced the melanin content in B16 melanoma cells and effectively inhibited mushroom tyrosinase activity [12].

Despite its long-established photoprotective role, there is evidence that melanin may also induce oxidative DNA damage in keratinocytes after UV exposure, and α-melanocyte-stimulating hormone (α-MSH) secreted from keratinocytes activates the melanocortin receptor 1 (MC1R) on the surface of melanocytes, which in turn triggers cAMP generation and PKA activation. It then translocates to the cell nucleus and phosphorylates CREB at Ser133, facilitating its binding with the CREB-binding protein, which enhances the expression of MITF and induction of downstream melanogenic genes [13,14,15]. MITF regulates the development of several cell types, including melanocytes, and is a key regulating transcription factor in melanocytes, responding to UVR [16,17,18]. CREB, a bZIP transcription factor, enables MITF-M expression to be responsive to elevated cAMP levels downstream from the MC1R via its ability to regulate MITF expression, which is regulated via MAPK signaling [19,20]. MAPK family proteins include extracellular signal-regulated kinase 1/2 (ERK1/2), c-Jun amino-terminal kinase (JNK) and p38. The activation of JNK directly phosphorylates CRTC3, thereby inhibiting nuclear translocation and suppressing UV-induced MITF expression and melanogenesis [21]. The activation of ERK induces the phosphorylation of MITF at Ser73, which leads to the ubiquitination and degradation of MITF [22,23]. In contrast to this pathway, the activation of ERK can lead to the phosphorylation of CREB, and the phosphorylated CREB then binds to the common motif of CRE in the MITF promoter region to up-regulate MITF [24]. MAPK may inhibit melanin formation through the inhibition of CREB phosphorylation and then the down-regulation of MITF expression. These results, suggesting that JNK regulates the ERK–CREB–MITF signaling pathway, play an important role in finding a new treatment strategy for skin-disease-related pigmentation diseases, such as vitiligo.

In our previous study, we identified that Heijingang purple potato (*Solanum tuberosum* L.) contained large amounts of anthocyanins, which were potential inhibitors of tyrosinase activity and possessed an anti-melanogenic effect, such as petanin, a major acylated anthocyanin [25]. However, the mechanism of the anti-melanogenic effect of petanin was unclear. Thus, petanin from *Solanum tuberosum* L. was used to study the tyrosinase activity and anti-melanogenesis in zebrafish, including the melanin inhibition rate, tyrosinase activity, distribution and arrangement of melanocytes, melanosome structure, oxidoreductase activity, network pharmacology, molecular docking, dynamic simulation and protein expression, mRNA transcription of melanogenesis-related signaling pathways, etc. These results are expected to provide a more comprehensive understanding of the mechanism of the anti-melanogenic effect of petanin and to promote the wider use of anthocyanins in pigmentation diseases.

## 2. Results and Discussion

### 2.1. Melanin Inhibition Rate

**Figure 1:**
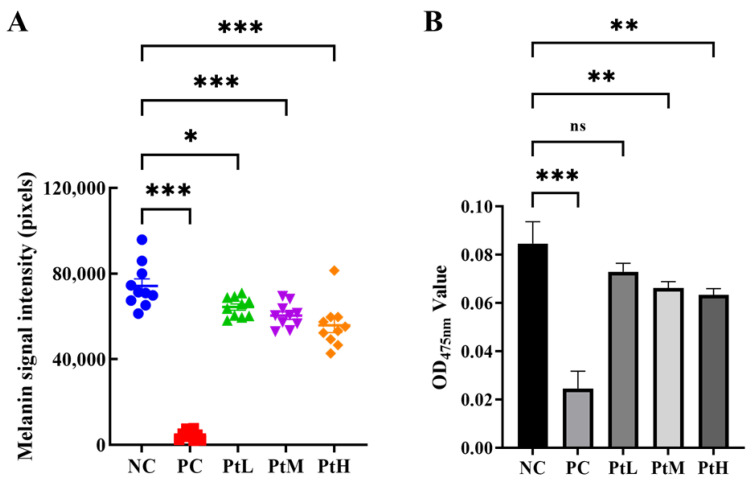
The comparison of the melanin (**A**) and tyrosinase (**B**) inhibition rate of petanin in zebrafish (NC: water; PC: 11 mM arbutin; PtL: 0.38 mM petanin; PtM: 0.80 mM petanin; PtH: 1.6 mM petanin; *: *p* < 0.05; **: *p* < 0.01; ***: *p* < 0.001; ns: not significant).

**Figure 2:**
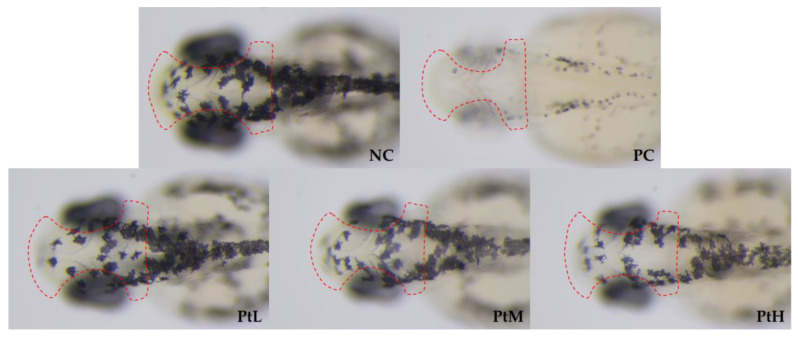
Typical images of the petanin inhibition of melanin production in zebrafish (NC: water; PC: 11 mM arbutin; PtL: 0.38 mM petanin; PtM: 0.80 mM petanin; PtH: 1.6 mM petanin; Scale: 1×; Zebrafish larvae: incubated for 45 h; Dotted red line: region of melanin signal intensity analysis in zebrafish head).

The effects of different concentrations of petanin on the melanin inhibition rate in zebrafish are shown in Figure 1A and Figure 2 and Table 1. Compared with the normal control (NC) group, petanin had a significant inhibitory effect on melanin production in zebrafish, but there was no significant difference among different concentrations. Compared with the arbutin group (PC), the maximum tolerated concentration of petanin was only 0.15% due to its complex molecular structure, large molecular weight and large polarity, and the melanin inhibition rate of 0.15% petanin was 24.86%, which was less than 93.52% of 0.30% arbutin.

### 2.2. Tyrosinase Activity in Zebrafish

The effects of different concentrations of petanin on tyrosinase activity in zebrafish are shown in Figure 1B and Table 1. Compared with the NC group, petanin significantly inhibited the tyrosinase activity of zebrafish, but there was no significant difference among the different concentrations. Compared with PC, petanin showed less tyrosinase inhibitory activity than arbutin, which may be due to its complex molecular structure and large molecular weight, making it difficult to penetrate zebrafish.

### 2.3. Structure and Distribution of Melanocytes and Melanosomes

**Figure 3:**
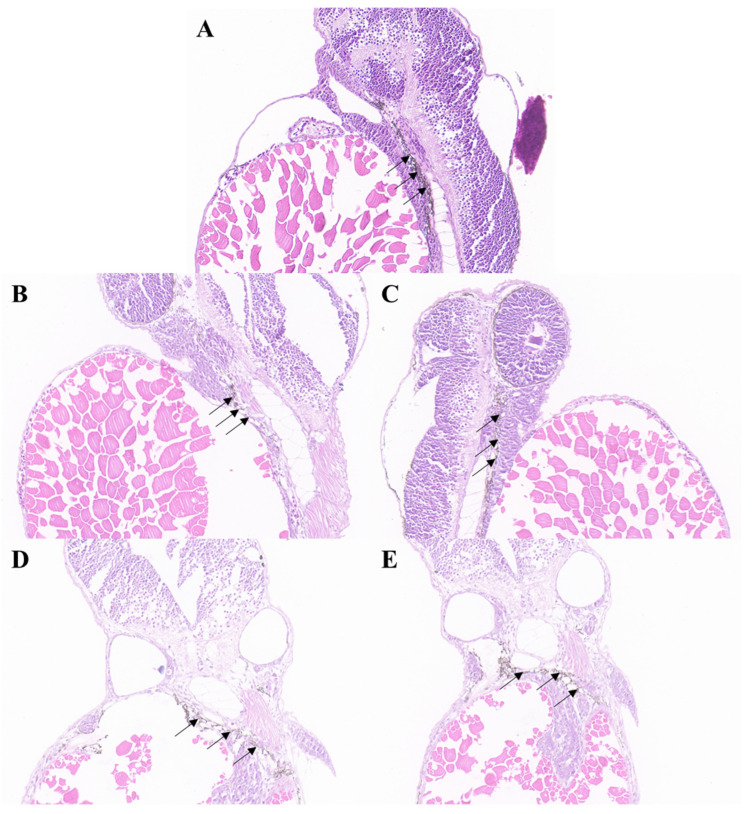
The effect of petanin on the distribution of melanocytes in zebrafish (HE, 20×). (**A**) NC group; (**B**) PC group; (**C**) PtL group; (**D**) PtM group; (**E**) PtH group; →: Melanocytes.

The results of hematoxylin and eosin (HE) staining of the effects of different concentrations of petanin on zebrafish melanocytes are shown in Figure 3. Melanocytes are found in the lower layer of the zebrafish epidermis (indicated with the arrow) and form obvious melanocyte clusters, with the epidermal cells showing a dense structure outside and the muscle cells showing a loose arrangement inside. Compared with the NC group, melanocytes in the low level and moderate level of petanin groups (PtL and PtM) showed different degrees of compact arrangement and stratification, while there were significantly fewer melanocytes in the PC and PtH groups, and they were loosely clustered [26]. However, other structures, such as iridocytes and yellow pigment cells, could not be observed under HE staining.

**Figure 4:**
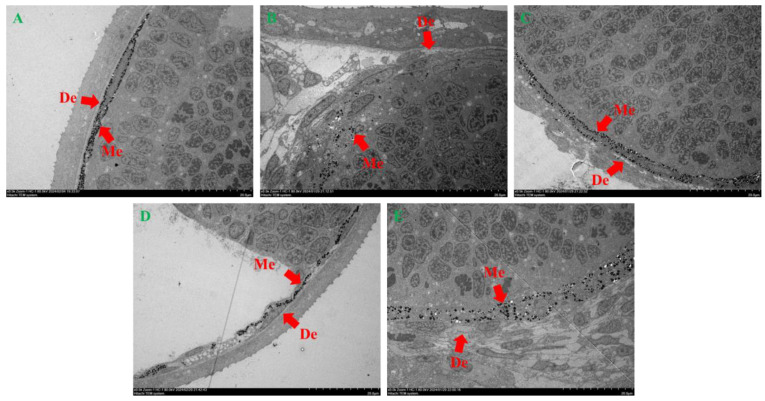
The effect of petanin on the arrangement of melanocytes in zebrafish (TEM, minimum scale value = 2.0 μm, De: dermis; Me: melanocyte. (**A**) NC group; (**B**) PC group; (**C**) PtL group; (**D**) PtM group; (**E**) PtH group).

**Figure 5:**
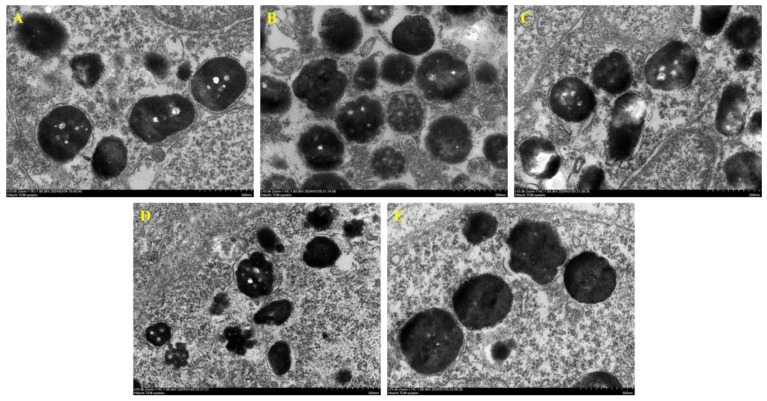
The effect of petanin on the structure of melanosomes in zebrafish (TEM, minimum scale value = 50 nm. (**A**) NC group; (**B**) PC group; (**C**) PtL group; (**D**) PtM group; (**E**) PtH group).

The results of TEM observation of different concentrations of petanin on zebrafish melanosomes are shown in Figure 4. The melanocytes in the NC group, PtL group and PtM group are arranged neatly to form local melanosomes or clusters, and some areas are manifested as inner and outer layers. However, the melanocytes in the PC and PtH groups are thinly arranged and obviously mixed with other types of cells, such as yellow melanocytes and iridocytes; therefore, they do not have an obvious melanin layer or cluster structure. Then, the melanosomes in melanocytes of each group are observed at the scale of 50 nm in Figure 5. There is no significant difference in the structure, distribution and number of melanosomes among the NC group, PtL group and PtM group; meanwhile, the structures of the melanosomes in the PC group and PtH group appear round, closely arranged and evenly distributed [26,27,28].

### 2.4. Effect of Petanin on Oxidoreductase Activity in Zebrafish

**Figure 6:**
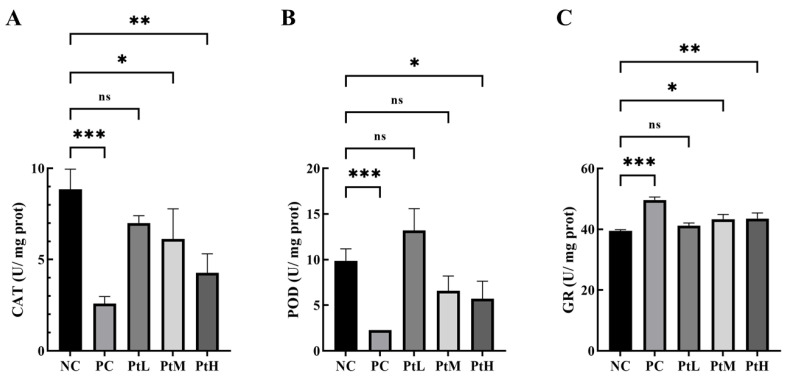
The effects of different treatments on oxidoreductase activity in zebrafish ((**A**) CAT (compared to NC group, ***: *p* < 0.001, **: *p* < 0.01, *: *p* < 0.05, ns: not significant), (**B**) POD (compared to NC group, ***: *p* < 0.001, *: *p* < 0.05, ns: not significant), (**C**) GR (compared to NC group, ***: *p* < 0.001, **: *p* < 0.01, *: *p* < 0.05, ns: not significant)).

The effects of different concentrations of petanin on oxidoreductases are shown in Figure 6. Compared with the CAT activity of the NC group, the results showed that the activities of the PC, PtM and PtH groups were significantly decreased, especially in the PC and PtH groups (*p* < 0.001). The results showed that the POD activities of the PC and PtH groups were significantly lower than that of the NC group (*p* < 0.05), but the activities of the PtL and PtM groups showed no significant difference. The results showed that the GR activities of the PC, PtM and PtH groups were significantly higher than that of the NC group, especially in the PC and PtH groups (*p* < 0.01). Oxidoreductase activity plays an important role in regulating oxidative stress during zebrafish development. Acute and subacute toxicity studies of zebrafish exposed to cadmium nanoparticles have shown that the activities of POD and CAT were significantly increased [29]. The toxic effects of short-term exposure to permethrin on zebrafish showed that induction of reactive oxygen species (ROS) accumulation could increase the activities of GST, GPx and POD and decrease the activities of SOD and CAT [30]. Zolamide and methoxomicillin could significantly increase the activities of CAT and POD in zebrafish [31]. Anthocyanins could significantly reduce the activities of CAT and GR but could significantly restore the activities of oxidoreductase [32]. The coenzyme Q10 and ellagic acid down-regulate the α-MSH signaling pathway and/or induce Nrf2/ARE-mediated depigmentation activity of antioxidant genes; phenolics reduce melanogenesis in B16F10 cells, and zebrafish indicate that oxidative stress has an important effect on melanogenesis [33,34,35]. In conclusion, petanin might have a positive effect on melanin production in zebrafish through its effect on the activities of CAT, POD and GR.

### 2.5. Network Pharmacological Analysis of the Anti-Melanogenic Effect of Petanin

**Figure 7:**
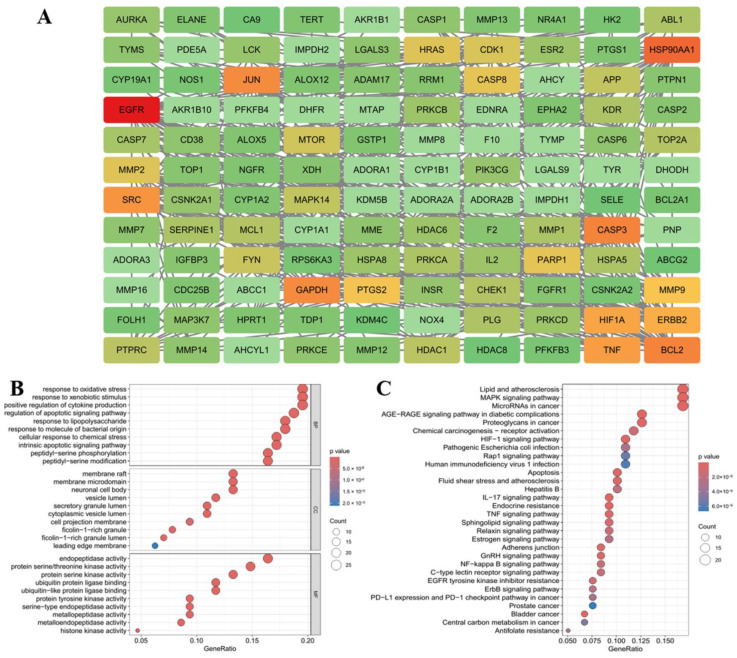
The network pharmacological analysis of the anti-melanogenesis effect of petanin ((**A**) important intersection targets; (**B**) important life processes; (**C**) key signaling pathways).

Tyrosinase was the rate-limiting enzyme of melanin production in melanocytes. Through gene expression, transcription, translation and post-translational modification in the endoplasmic reticulum and Golgi, a crystal structure of two copper ions bounded with three histidine residues responsible for the catalytic activity of tyrosinase was formed, which was transported to the melanosomes to play catalytic roles, such as oxidation, amination and pre-oxygenation, at the active site to promote melanin production and pigmentation [8,36,37,38]. In zebrafish, petanin inhibited tyrosinase activity and melanin production in vivo, and it had a significant effect on oxidoreductase activity [25]. Therefore, network pharmacology tools were used to analyze the targets and signaling pathways related to the properties of petanin, its degradation products and melanin production. The targets of petanin and its degradation products were obtained through SwissTargetPrediction; 4122 targets were obtained through Genecards, Disgenet, OMIM and other databases, and 128 intersection targets were obtained via Venn analysis, as shown in Figure S1. The PPI network of 128 targets was explored using string data, and the key targets of petanin and its degradation products affecting melanin production were identified based on CytoNCA and Cytohubba analysis, for instance, EGFR, HSP90AA1, CASP3, BCL2, JUN, GAPDH, SRC, ERBB2, MAPK14 and TYR, as shown in Figure 7A and Figure S2.

GO enrichment analysis showed that the core targets were mainly enriched in biochemical processes, such as the oxidative stress response, exogenous stimulus response, positive regulation of cytokines, etc. (Figure 7B). KEGG enrichment analysis revealed that the core targets were mainly enriched in blood lipids, atherosclerosis, MAPK signaling pathway, etc. (Figure 7C). Melanogenesis was regulated via the MC1R/α-MSH, PI3K/AKT, PKA/CREB and MAPK signaling pathways; in particular, MAPKs regulated CREB/MITF, a key protein in melanogenesis [39]. The activation of p38 MAPK played an important role in the process of placental total lipid-induced B16F10 melanogenesis through the up-regulation of tyrosinase expression [40]. Ganoderma lucidum polysaccharide could inhibit UVB-activated PKA and MAPK signaling pathways and down-regulate the expression of genes related to melanin production [41]. The inhibitory effect of eupafolin on melanogenesis showed that it could down-regulate the expression of Akt, up-regulate the phosphorylation of ERK1/2 and p38 MAPK and down-regulate the expression of tyrosinase and related proteins through the down-regulation of the expression of cAMP and MITF [42]. Therefore, the key proteins in the MAPK signaling pathway that regulate melanogenesis were screened, including ERK1/2, p38, JNK and PKA, and their potential target activities were simulated using a molecular docking platform to elucidate the anti-melanogenesis mechanism of petanin.

### 2.6. Molecular Docking and Dynamic Simulation of Petanin with Key Signaling Pathway Proteins

**Figure 8:**
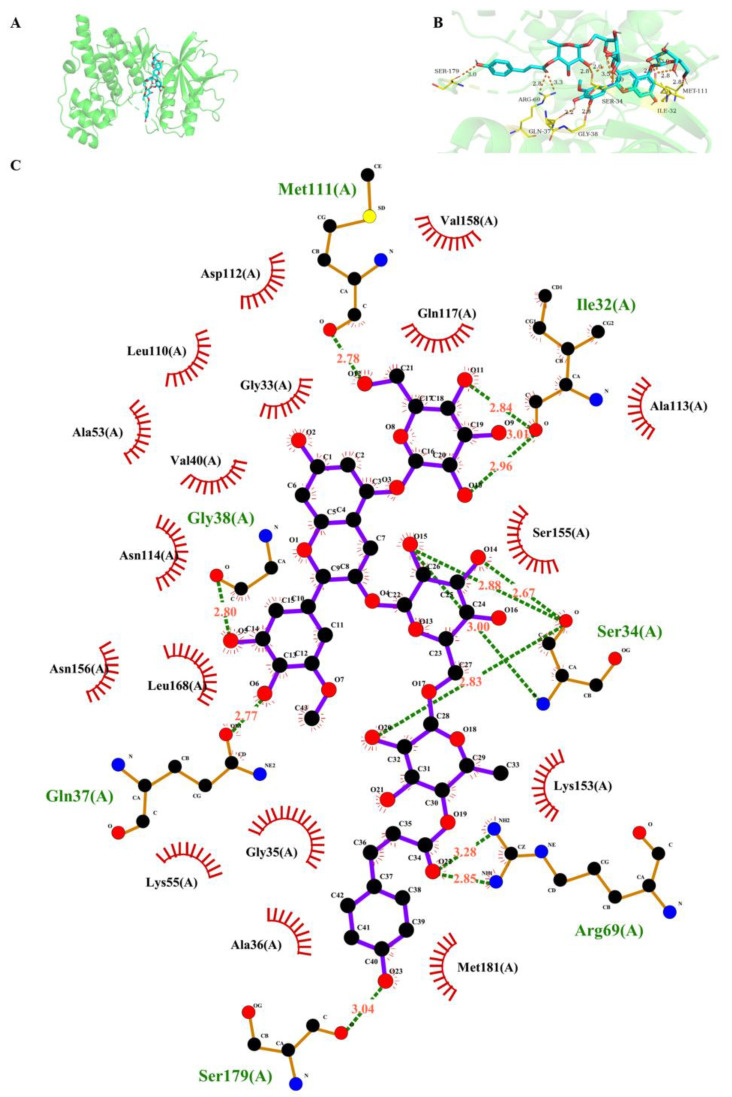
The molecular docking of petanin with JNK ((**A**) 3D structure; (**B**) 3D diagram of hydrogen bonds; (**C**) 2D diagram of hydrogen bonds).

The results of the molecular docking of petanin with key proteins involved in the regulation of melanogenesis in the MAPK signaling pathway are shown in Figure 8 and Figure S3 and Table S1. Petanin had the best affinity with JNK and abundant hydrogen bond binding sites, which were located in the molecular pocket of JNK but on the surface of ERK, leading to the difference in binding energy (Figure 8A). The hydrogen bond site between petanin and JNK was mainly at the acyl sugar of C8-O4-C22, which was clearly different from ERK at the sugar group of C3-O3-C16, thus affecting the stability of the complex (Figure 8B,C).

**Figure 9:**
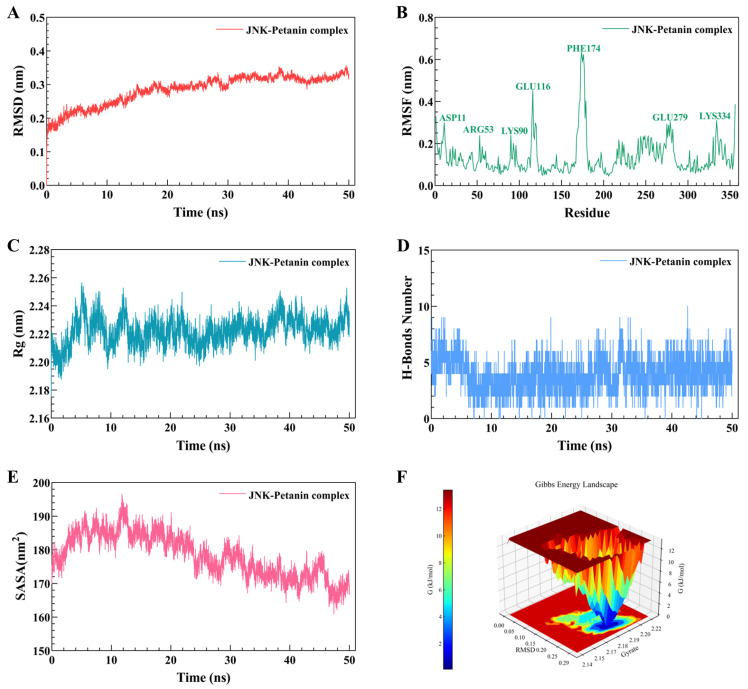
The molecular dynamics’ simulation of petanin–JNK complex ((**A**) RMSD; (**B**) RMSF; (**C**) Rg; (**D**) H-bonds number; (**E**) SASA; (**F**) Gibbs energy landscape).

The molecular dynamics’ simulations of petanin and JNK are shown in Figure 9. The RMSD fluctuation curves of the petanin–JNK complex reach a stable state within 50 ns without large fluctuations, with a fluctuation range of 0.2–0.3 nm, indicating that petanin could rapidly form a stable complex with JNK. The RMSF results showed that the amino acid residues in the petanin–JNK complex fluctuated around the amino acid residues at the ASP11, ARG53, LYS90, GLU116, PHE174, GLU279 and LYS334 sites, especially PHE174 with an amplitude of 0.6 nm, which is a normal fluctuation, caused by the binding of the constituent small molecules to the protein. The results of Rg showed that the Rg curves of petanin–JNK all fluctuated in the range of 2.20–2.24 nm, and the fluctuation amplitude was only 0.04 nm, indicating that the complex forms a tight and stable structure. The number of hydrogen bonds between petanin and JNK was five, which ensured the stability of the complex. The results of the solvent-accessible surface area (SASA) showed that the petanin–JNK complex had a relatively stable solvent contact area; the fluctuation range was 170–190 nm^2^, and the range was only 20 nm^2^, which indicated that the structure and solvation effect of the complex was very stable. The Gibbs free energy plots showed that petanin–JNK exhibited a single and broad energy cluster, indicating the formation of a stable binding between the protein and the ligand in this complex.

### 2.7. Effect of Petanin on the Expression of Proteins Related to Melanin Production in Zebrafish

**Figure 10:**
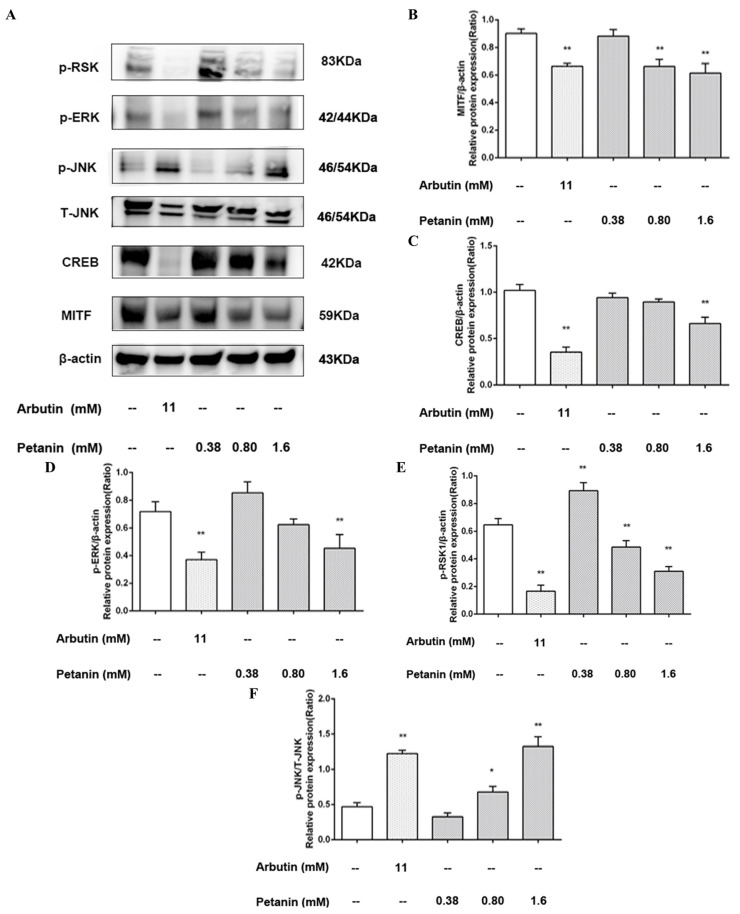
The effects of petanin on the ERK/CREB/MITF signaling pathway ((**A**) protein expression level; (**B**) MITF; (**C**) CREB; (**D**) p-ERK; (**E**) p-RSK1; (**F**) p-JNK; *: *p* < 0.05; **: *p* < 0.01).

The effects of different concentrations of petanin on the expression levels of proteins involved in the melanogenesis signaling pathways in zebrafish are shown in Figure 10, and the expression levels of β-actin, representing the internal reference protein expression level of each treatment group, were in good agreement. Petanin inhibited CREB and MITF, the key proteins of melanin production, in different concentrations compared with the NC group (Figure 10B,C). The PtH group showed a very significant difference in MITF (*p* < 0.01) and no significant difference with the PC group, while the inhibition of CREB was weaker than that of the PC group. Decursin inhibited melanogenesis via the down-regulation of MITF through the PKA/CREB pathway [39]. Glabridin significantly down-regulated the transcription and/or protein expression of MC1R, MITF, TYR, TRP-1 and TRP-2 in B16 cells [43]. Calycosin blocked CREB, and the p38 MAPK-mediated signaling pathways were validated by PKA and p38 inhibitors [44]. Schisandrin B down-regulated the expression levels of TYR, MITF, TRP-1 and TRP-2 via intervention in the expression of p38, ERK, JNK and CREB [45]. Fargesin significantly reduced the expression of the MITF protein and inhibited the interaction of PKA/CREB and the activation of p38 MAPK [46]. On the contrary, imperatorin and isoimperatorin promoted melanogenesis through the down-regulation of ERK, PI3K, GSK-3β and β-catenin and the up-regulation of PKA/CREB/MITF signaling pathway [47]. Hence, the anti-melanogenic effect of petanin might be mediated via the regulation of tyrosinase expression through the CREB/MITF signaling pathway.

Furthermore, the expression of MAPK-related proteins downstream of the CREB/MITF signaling pathway was investigated (Figure 10D,E). Compared with the NC group, different concentrations of petanin could inhibit p-ERK and p-RSK to different degrees; in particular, the PtH group showed a significant difference (*p* < 0.01) and a dose-dependent manner. In other studies completed in this area, the anti-melanogenic effects of natural substances have been further investigated. It was identified that Mannosylerythritol lipids significantly inhibited the expression of typical melanogenic enzyme-related genes, such as *tyr*, *trp-1* and *trp-2*, via inhibition of the ERK/CREB/MITF signaling pathway [48]. In addition, astaxanthin was shown to inhibit the stem cell factor (SCF)-induced expression of MITF, TYR and endothelin receptor B, and it down-regulated the phosphorylation of CREB, which affected the c-KIT/Shc/Raf-1/ERK/RSK/CREB signaling axis and inhibited the phosphorylation of MSK1 to affect the c-KIT/p38/MSK1 signaling axis [49]. It was also demonstrated that French maritime pine bark extract was resistant to the UVB-induced up-regulation of MSK1 and CREB phosphorylation, and it had no significant effect on phosphorylated p38 and JNK, which were independent of the ERK/RSK/CREB pathway [50]. Liver X receptor TO901317 inhibited the expression of TYR, TRP-1 and TRP-2 without affecting tyrosinase activity and the expression of MITF and PKA; however, it accelerated the degradation of MITF and was associated with the phosphorylation of the MEK/ERK/RSK1 signaling cascade [51]. In another study, gallic acid was shown to decrease the expression of MITF, tyrosinase, TRP-1 and dopachrochrome isomerase, and it promoted the phosphorylation of Akt and MEK/ERK [52]. A study has also demonstrated that Sargaquinoic acid inhibited the expression of TYR, TRP-1 and TRP-2, with the effect of improving the levels of pigmentation, decreasing cAMP accumulation and inhibiting CREB to down-regulate MITF and increasing the phosphorylation of ERK1/2 and MITF to induce the proteasomal degradation of MITF [53]. Consequently, this study demonstrated that petanin might down-regulate the expression of CREB/MITF-related melanogenic proteins through the inhibition of phosphorylation of the ERK/RSK-related signaling cascade.

Finally, the expression of p-JNK and T-JNK proteins was investigated (Figure 10F). In this study, it was identified that PtM, PtH and PC significantly up-regulated the phosphorylation of JNK compared with NC; PtH and PC showed a significant difference, and petanin showed a dose-dependent manner. Another study demonstrated that *Nymphaea nouchali* flower extract inhibited melanogenesis through the regulation of the cAMP/CREB/MAPK/MITF signaling pathway and the proteasomal degradation of tyrosinase [54]. In particular, it is possible that the up-regulation of JNK phosphorylation might mediate the anti-melanogenesis effect. The natural organic compound Sesamol was shown to decrease melanin production through decreasing the cAMP accumulation, tyrosinase activity and expression of TYR, Trp-1 and Trp-2, MITF and MC1R, and it was associated with enhanced phosphorylation of p38 MAPK and JNK [55]. In addition, Paeonol reduced melanin production through the down-regulation of the expression of MITF and TYR mRNA and the phosphorylation of CREB; however, the anti-melanogenic effect was reversed with the inhibition of JNK/SPARK [56]. JNK activity was shown to inhibit melanogenesis via the phosphorylation of CRTC3 and the blocking of its nuclear translocation and interfering with the CRTC3-dependent MITF-M expression [57]. In contrast, ERK1/2 catalyzed the phosphorylation of CRTC3 at SER391 to increase its interaction with calcineurin, thereby hydrolyzing the phosphorylated group on the binding site of CRTC3 to 14-3-3 protein. In brief, phosphorylated JNK might play an important role in the inhibitory effect of petanin on melanin production in zebrafish, which may be due to the negative regulation of the ERK/RSK/CREB/MITF cascade signaling pathway.

### 2.8. Effect of Petanin on Transcription of mRNAs Related to Melanogenesis in Zebrafish

**Figure 11:**
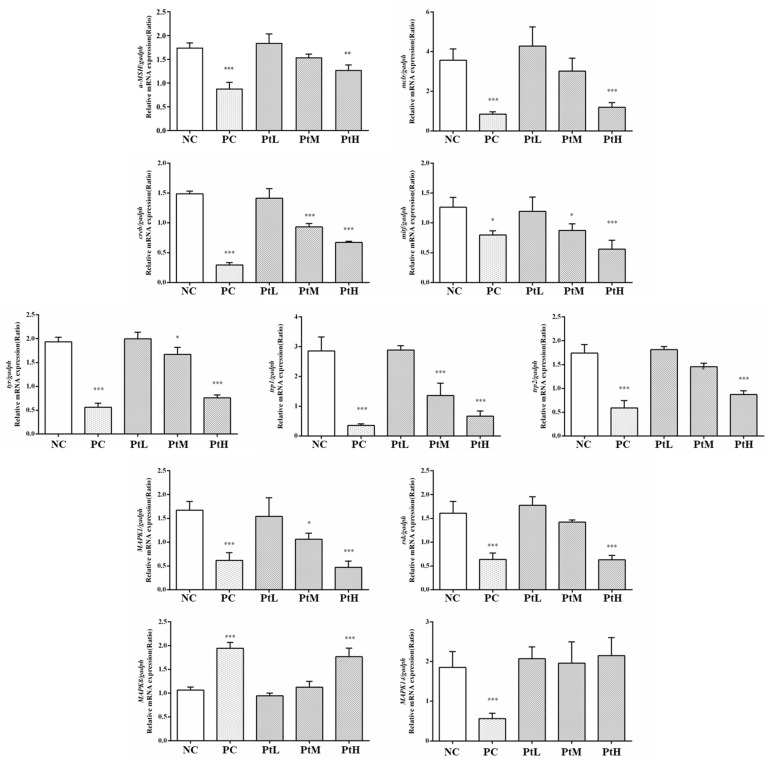
The effect of petanin on the transcription of mRNA related to melanogenesis in zebrafish (NC: water; PC: 11 mM arbutin; PtL: 0.38 mM petanin; PtM: 0.80 mM petanin; PtH: 1.6 mM petanin; *: *p* < 0.05; **: *p* < 0.01; ***: *p* < 0.001).

The effects of different concentrations of petanin on mRNA transcription related to melanin production in zebrafish are shown in Figure 11. Compared with the NC group, the transcription levels of *α-MSH*, *mc1r*, *creb*, *mitf*, *tyr*, *trp1* and *trp2* were significantly down-regulated in the PtH group and PC group in a dose-dependent manner (*p* < 0.01). Interestingly, the effects of petanin on the MAPK-signaling-pathway-related genes *erk*, *p38*, *rsk* and *jnk1* were specific. For example, petanin could significantly up-regulate the transcription level of *jnk1* and inhibit the transcription of *erk* and *rsk*, but it had no effect on *p38*. Collectively, these results validate the critical role of JNK in the regulation of melanin production by petanin in zebrafish. JNK inhibited melanogenic production through interference with the CREB-regulated transcription-coactivator-3-dependent MITF expression, and forskolin treatment significantly increased the transcription levels of *mitf* and *tyr* genes. Meanwhile, in another study, Ro31-8220, a CREB/CRTC inhibitor, was shown to be able to protect against the increased transcription [21]. It has also been identified that maclurin can enhance the transcription levels of mitf, trp-1, trp-2 and tyr and activate the cAMP/PKA/CREB and p38 MAPK/CREB signaling pathways to promote melanogenesis [58]. Cordycepin was shown to decrease the mRNA transcription levels of *tyr*, *trp-1* and *trp-2* while inhibiting the α-MSH- and IBMS-induced melanogenesis via the down-regulation of CREB/MITF and the activation of PI3K/Akt and ERK [59]. In another study, it was shown that methyl-2-acetylamino-3-(4-hydroxy-3,5-dimethoxybenzoylthio) propanoate reduced *tyr*, *trp-1* and *trp-2* mRNA transcription, but it had no effect on the phosphorylation of p38 MAPK, JNK and AKT [60]. In this study, the downstream of MITF regulation was not in transcription but translation, i.e., the ERK signaling pathway mediated the MITF proteasomal degradation. Eventually, petanin might achieve its anti-melanogenic effect by regulating mRNA transcription related to melanogenesis and the expression of related regulatory proteins in zebrafish.

## 3. Materials and Methods

### 3.1. Samples and Reagents

Petanin was isolated and purified from *Solanum tuberosum* L., which was identified via NMR, spectroscopic, mass spectrometric and chromatographic analyses and comparison with the compound reported in the literature with a purity of 99.43% (Figures S4–S6). Arbutin (CAS: 497-76-7, Product code: A106856-25g, purity ≥ 98%) was purchased from Shanghai Aladdin Biochemical Technology Co., Ltd. (Shanghai, China).

### 3.2. Melanin Inhibition Rate in Zebrafish

The animal studies conducted in this research were approved by the Institutional Animal Care and Use Committee of Hangzhou Hunter Bio Co., Ltd. (Hangzhou, China) (IACUC-2023-7602-01). AB-strain zebrafish were procured from a commercial supplier and maintained under controlled conditions with a 14/10 h light/dark cycle at 28.5 °C. Embryos were obtained through natural spawning and collected in embryo medium. Zebrafish embryos at 6 h post-fertilization (hpf) was randomly assigned to individual wells of 6-well plates, each containing 30 fish with a volume of 3 mL. Different concentrations of petanin groups (PtL: 0.38 mM petanin; PtM: 0.80 mM petanin; PtH: 1.6 mM petanin) along with a normal control group (NC: water) and a positive control group (PC: 11 mM arbutin) were administered to the zebrafish embryos. The plates were then incubated at 28 °C under light-avoiding conditions for a duration of 45 h. Subsequently, 10 zebrafish from each experimental group were randomly selected and imaged using a dissecting microscope for further analytical purposes using ImageJ 1.37v software to quantify the intensity of melanin signals in their heads [61,62,63]. According to Equation (1), whether the sample had a melanin inhibition rate was calculated and judged.

(1) $$Melanin\;inhibition\;rate\;(\%)=\frac{S\;(Normal)-S\;(Sample)}{S(Normal)}*100\%,$$

### 3.3. Tyrosinase Activity Assay in Zebrafish

The samples were dissolved in water, with the volume of each well set as 3 mL (N = 3), and the normal control group was established. The cells were incubated at 28 °C in the dark for 45 h. The protein concentration of each experimental group was determined with a BCA protein concentration assay kit (P0010, Beyotime Biotech. Inc., Shanghai, China). The 250 μg total protein of zebrafish samples was added to 1 mM levodopa solution at a volume ratio of 1:1 and mixed. The liquid was transferred to a 96-well plate at 200 μL/well, and the absorbance value was measured at a wavelength of 475 nm using a microplate reader. The tyrosinase inhibition rate of the samples was calculated according to Equation (2).

(2) $$Inhibition\;rate\;of\;tyrosinase\;(\%)=\frac{OD475(Normal)-OD475\;(Sample)}{OD475(Normal)}*100\%,$$

### 3.4. Detection of CAT, POD and GR Contents in Zebrafish Larvae

The zebrafish larvae were washed three times with phosphate-buffered saline (PBS) and subsequently transferred to ice-cold EP tubes. Mechanical homogenization was performed on the tissue lysate. Following the manufacturer’s instructions, the levels of glutathione reductase (GR), catalase (CAT) and peroxidase (POD) in the zebrafish larvae were determined using their respective detection kits (GR: S0055 (Beyotime Biotech. Inc., Shanghai, China); CAT: BC0205 (Beijing Solarbio Science & Technology Co., Ltd., Beijing, China); POD: BC0095 (Beijing Solarbio Science & Technology Co., Ltd., Beijing, China)).

### 3.5. HE Staining

The zebrafish embryos were collected and placed in EP tubes. Subsequently, they underwent three 5 min washes with phosphate-buffered saline (PBS). Then, they underwent fixation using tissue fixative (G1101; Servicebio, Wuhan, China) at room temperature and were incubated overnight in darkness. The wax blocks containing the embedded samples were sectioned using a Leica paraffin microtome (RM2016, Leica Microsystems (Shanghai) Trading Co., Ltd., Shanghai, China). These sections were stained with hematoxylin and eosin (HE) stains and sealed using neutral resin and coverslips. Finally, these sections were examined under a Nikon microscope (Eclipse E100, Nikon Inc., Tokyo, Japan).

### 3.6. TEM

Transmission electron microscopy was performed as previously described with some modifications. Zebrafish embryos were collected and fixed with 2.5% glutaraldehyde at 4 °C for 2 h. After a series of ethanol dehydration steps, embedding, polymerization, sectioning and staining were performed. Next, the 60~80 nm thick sections were stained using uranyl acetate and citric acid (Leica EM UC7, Leica Microsystems (Shanghai) Trading Co., Ltd., Shanghai, China). Finally, images were taken and visualized via electron microscopy (HITACHI HT 7800 120kv; Tokyo, Japan).

### 3.7. Network Pharmacological Analysis

Network pharmacological analyses of the anti-melanogenic effects of petanin and its degradation products (norpetanin, lyciruthephenylpropanoid D/E and 4-*O*-(*p*-coumaryl)) were conducted. The target sites of five compounds were predicted using SwissTargetPrediction. The databases of Genecards, Disgenet and OMIM were used and searched using the keyword “melanoma”. Genecards was selected with a relevance score ≥ 1, and Disgenet was screened using Score_gda ≥ 0.1. Venn analysis was used to interpose the targets of components and the targets of melanoma. PPI was explored using the String database, where the selected screening confidence interval was 0.7. The network topology value information was calculated using CytoNCA, and the heat map of PPI was drawn according to Degree. TOP20 targets were calculated according to Degree using Cytohubba. KEGG and GO enrichment analyses were performed for intersection targets and TOP20 targets, respectively.

### 3.8. Molecular Docking and Molecular Dynamics’ Simulation

The interaction between petanin and key target proteins was analyzed using AutoDockTools 1.5.6 and PyMOL (Version 2.3.4) software. The 2D structures of petanin were drawn using ChemDraw and converted into 3D structures with minimal energy using Chem3D (Version 20.0.0.41) software. The 3D structures of the proteins, including ERK (PDBID: 5KE0), P38 (PDBID: 3ZS5), PKA (PDBID: 1CX4) and JNK (PDB ID: 4QTD), were obtained from the Protein Database (PDB, http://www.rcsb.org/ (accessed on 2 March 2024)). Ligand residues were removed from the proteins using PyMOL software, while the receptor protein was hydrogenated and saved in PDBQT format using AutoDockTools 1.5.6 software. Both compounds and receptor proteins were stored in PDBQT format for further analysis. Active pocket sites were defined across the entire protein structure to identify potential binding regions. Finally, AutoDock Vina was employed to dock and determine the optimal construct based on the active amino acid site information derived from the literature.

The virtual molecular docking results were further investigated through explicit solvent molecular dynamics’ (MDs) simulations. The GROMACS v.2019.4 package was employed to perform a 50 ns MD simulation of protease–ligand complexes, following the previously described method. After energy minimization and equilibration of all systems, an unrestrained MD production run was conducted for 50 ns with a time step of 2 fs, saving the structure coordinates every 10 ps. Subsequently, the trajectories obtained from the completed 50 ns MD simulation were utilized for various dynamic analyses, including root mean square deviation (RMSD), root mean square fluctuation (RMSF), radius of gyration (Rg), number of hydrogen bonds, Gibbs free landscape analysis and secondary structural analysis using different built-in scripts in GROMACS.

### 3.9. Western Blot

Thirty zebrafish larvae were collected and washed three times with PBS and subsequently transferred to ice-cold EP tubes. After removing PBS, pre-chilled RIPA Lysis buffer (Beyotime, Shanghai, China) containing 1% phenylmethylsulfonyl fluoride (PMSF; Beyotime, Shanghai, China) was added to each well. The tissue was homogenized using mechanical homogenization. Then, the tissue was incubated on ice for 30 min. Centrifugation was performed at 12,000 rpm at 4 °C for 15 min. The resulting supernatant was collected for subsequent experiments. The total protein concentration was determined using the BCA protein detection kit (Beyotime, Shanghai, China) for the comprehensive quantification of the protein. The prepared samples were loaded onto sodium dodecyl sulfate–polyacrylamide gel electrophoresis (SDS-PAGE) and transferred to polyvinylidene fluoride (PVDF). Membranes were blocked in a solution of 5% skim milk for 1 h before being washed three times with Tris-buffered saline with Tween-20 (TBST), followed by overnight incubation at 4 °C with primary antibodies: rabbit anti-JNK (1:1000; Huabio, Hangzhou, China), rabbit anti-CREB (1:500; Huabio, Hangzhou, China), rabbit anti-β-actin (1:1000; Huabio, Hangzhou, China), rabbit anti-MITF (1:500; ABclonal, Wuhan, China), rabbit anti-phospho-RSK (p-RSK) (1:500; ABclonal, Wuhan, China), rabbit anti-phospho-JNK (p-JNK) (1:400; Abcam, Waltham, MA, USA) and rabbit anti-phospho-ERK (p-ERK) (1:500; CST, Danvers, MA, USA). Subsequently, the membranes were washed three times using TBST and then incubated with horseradish peroxidase-conjugated secondary antibody (1:1000; Beyotime Biotechnology, Shanghai, China) for 2 h. After another three washes with TBST, the protein bands were detected using an ECL reagent (Beyotime, Shanghai, China). The relative gray-scale values were analyzed using Image J software.

### 3.10. Gene Expression Analysis

The total RNA of zebrafish embryos was extracted after incubation with varying petanin concentrations and subsequently reverse transcribed to form cDNA. SYBR Premix Ex Taq (2×) (Servicebio, Wuhan, China) was used for the qPCR, and GADPH was used as the internal control. Table S2 (Supplementary Materials) shows the sequence of primers used in this study.

### 3.11. Statistical Analysis

All data were expressed as the mean ± SEM from three independent experiments. Statistical analysis was performed using one-way analysis of variance (ANOVA) using GraphPad Prism (version 9.5.0; GraphPad Software, Boston, MA, USA).

## 4. Conclusions

In this study, both the anti-melanogenic effect and the inhibition of tyrosinase activity of petanin was demonstrated. Firstly, petanin was able to significantly affect the activities of the oxidation-reduction enzyme and regulate the redox equilibrium in zebrafish, mainly manifested as a decrease in CAT and POD enzyme activities and an increase in GR enzyme activity. Secondly, network pharmacology, molecular docking and molecular dynamics’ simulations showed that petanin was screened and enriched for TYR targets and MAPK signaling pathways and suggested that the form of the petanin-JNK complex may be critical for its anti-melanogenic effect. Finally, petanin affected the protein expression and mRNA transcription of melanogenesis-related signaling pathways to be regulated via the CREB/MITF/TYR pathway, and the MAPK signaling pathway played an important role. The ERK/RSK signaling cascade could activate CREB/MITF to promote melanin production, in which the phosphorylations of p38 and ERK were positive, and the phosphorylation of JNK was negative. In particular, p-JNK might negatively regulate the ERK/CREB/MITF signaling pathway to affect the expression of tyrosinase protein and related transcription factors (Figure S7). Consequently, the anti-melanogenic effect of petanin might be based on the inhibition of tyrosinase activity and the down-regulation of tyrosinase expression. However, anthocyanins are widely believed to have low bioavailability, with the majority of nutrition studies reporting peak plasma concentrations ranging from 1 to 120 nmol L^−1^ and urinary recovery at <1% of intake, with the levels of excretion frequently reported as low as 0.005% [64]. Therefore, the mechanism of the anti-melanogenesis action of petanin should be investigated, focusing in future on drug availability and metabolomics to clarify its active groups and metabolic pathways.

## Figures and Tables

**Table 1:** The melanin inhibition rate of petanin in zebrafish (N = 10/30).

Sample	Melanin Inhibition Rate	Inhibition Rate of Tyrosinase	*p*-Value
NC	-	-	-
PC	93.52%	70.59%	˂0.001
PtL	13.26%	14.12%	˂0.05
PtM	18.64%	17.65%	˂0.01
PtH	24.86%	25.88%	˂0.001

## Data Availability

The original contributions presented in the study are included in the article/Supplementary Materials, further inquiries can be directed to the corresponding author.

## Supplementary Materials

The supporting information can be downloaded at: https://www.mdpi.com/article/10.3390/ijms25115939/s1.
