# Supplementary material for: Petanin Potentiated JNK Phosphorylation to Negatively Regulate the ERK/CREB/MITF Signaling Pathway for Anti-Melanogenesis in Zebrafish
Source: Int J Mol Sci. 2024 May 29;25(11):5939. doi: 10.3390/ijms25115939 (PMC11173099; doi:10.3390/ijms25115939)
Supplement: Supplementary file 1 [file ijms-25-05939-s001.zip › ijms-2978886-supplementary.pdf]

SUPPLEMENTARY MATERIAL

Petanin Potentiated JNK Phosphorylation to Negatively Regulate the ERK/CREB/MITF Signaling Pathway for Anti-Melanogenesis in Zebrafish

Jian Ouyang [1,2,3], Na Hu [1] and Honglun Wang [1,2,*]

[1] Qinghai Provincial Key Laboratory of Tibetan Medicine Research and CAS Key Laboratory of Tibetan Medicine Research, Northwest Institute of Plateau Biology, Xining 810008, China; ygzjj@126.com (J.O.); huna@nwipb.cas.cn (N.H.)
[2] Huzhou China-Science Innovation Centre of Plateau Biology, Huzhou 313000, China
[3] University of Chinese Academy of Sciences, Beijing 100049, China
* Correspondence: hlwang@nwipb.cas.cn

List of contents

Figure S1. VENN diagram of intersection of targets related to melanin and compounds((**A**) screening of OMIM targets, (**B**) intersection of compound targets and melanin targets)

Figure S2. Top 20 of Melanogenesis related targets intersect with targets of compounds

Figure S3. Molecular docking of petanin with ERK, p38 and PKA ((A) 3D structure), (B) 3D diagram of hydrogen bonds), (C) 2D diagram of hydrogen bonds)

Figure S4. HPLC and spectrogram of petanin (purity ≥99.43%)

Figure S5. ^{1}H NMR and structure of petanin

Figure S6. Mass spectrum of petanin from liquid chromatography-mass spectrometry

Figure S7. Effect of petanin on ERK/CREB/MITF signaling pathway

Table S1. Molecules docking of petanin binding energy and hydrogen bonds with key signaling pathway protein

Table S2. Primers for mRNA associated with melanogenesis in zebrafish

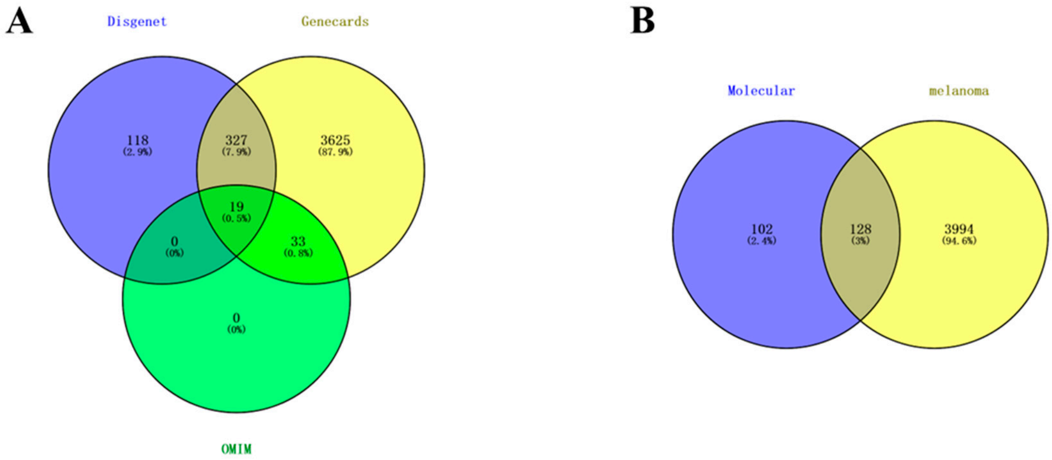

Figure S1. Venn diagram of intersection of targets related to melanin and compounds((**A**) Screening of OMIM targets, (**B**) Intersection of compound targets and melanin targets)

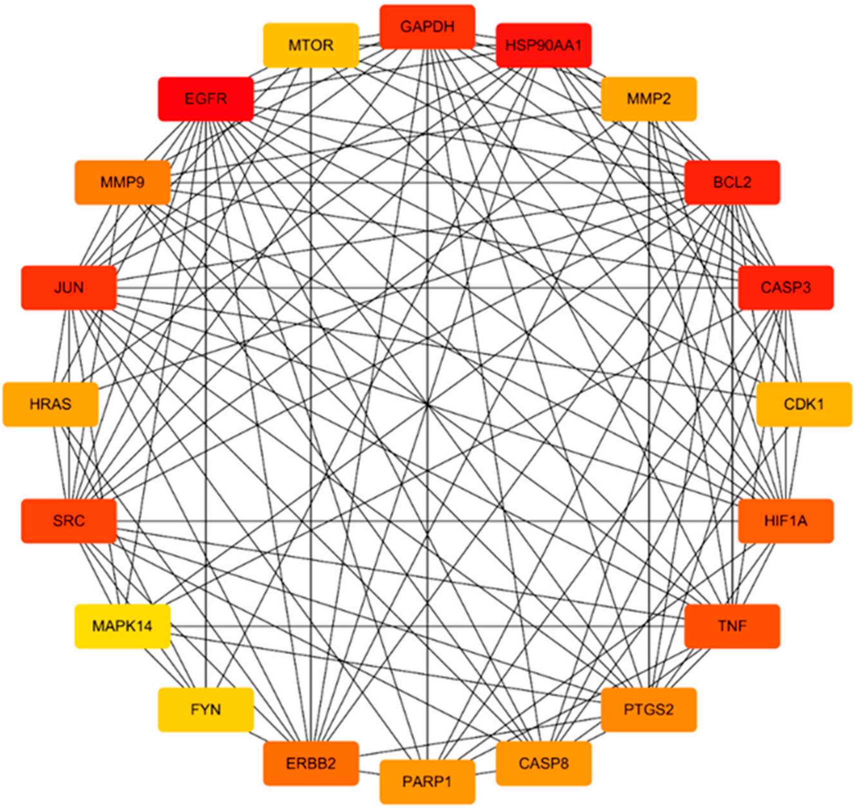

Figure S2. Top 20 of Melanogenesis related targets intersect with targets of compounds

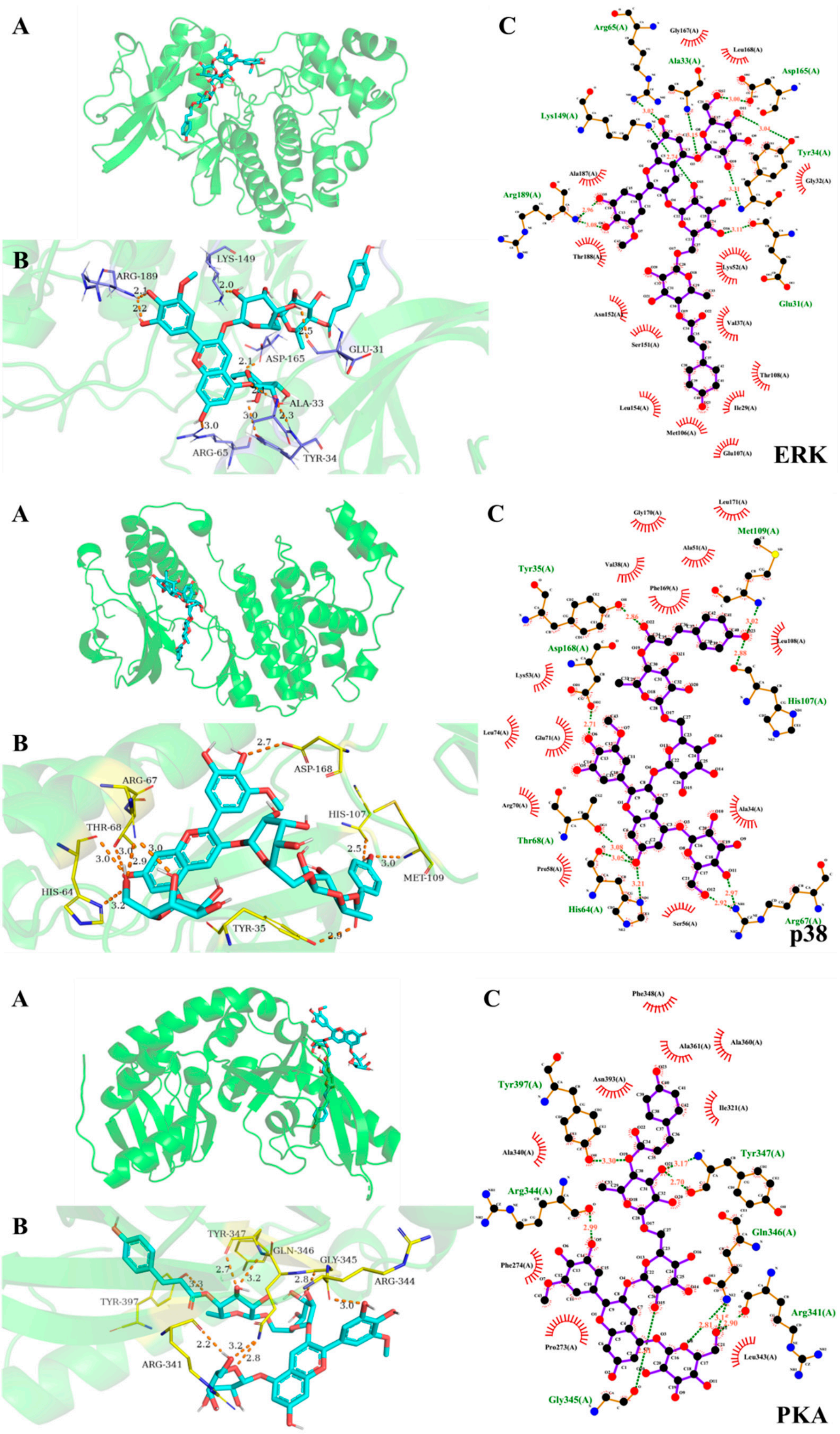

Figure S3. Molecular docking of petanin with ERK, p38 and PKA ((A) 3D structure), (B) 3D diagram of hydrogen bonds), (C) 2D diagram of hydrogen bonds).

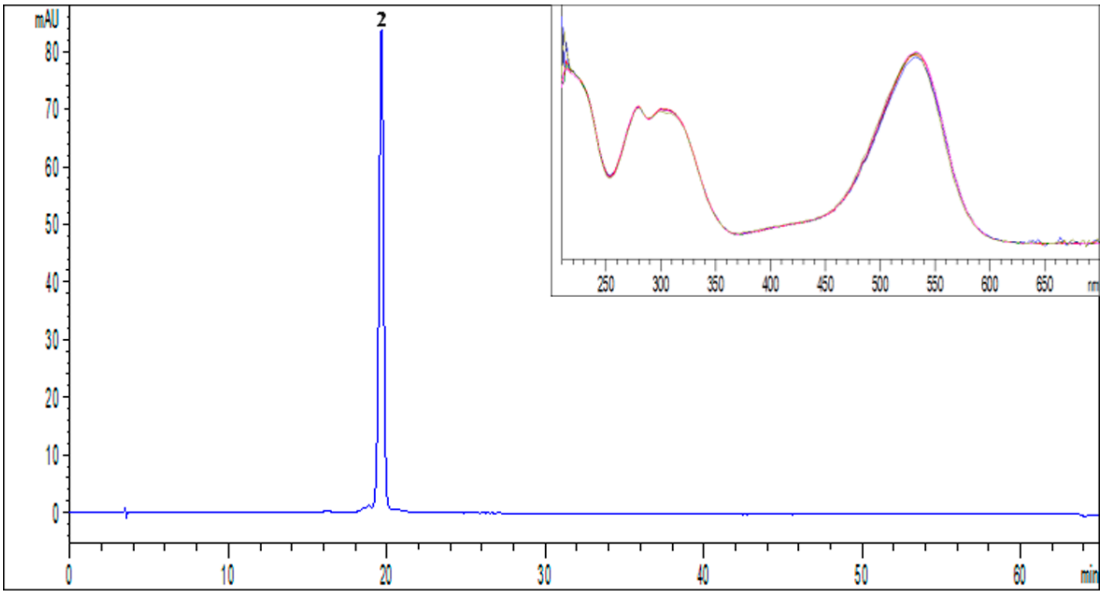

Figure S4. HPLC and spectrogram of petanin (purity ≥99.43%)

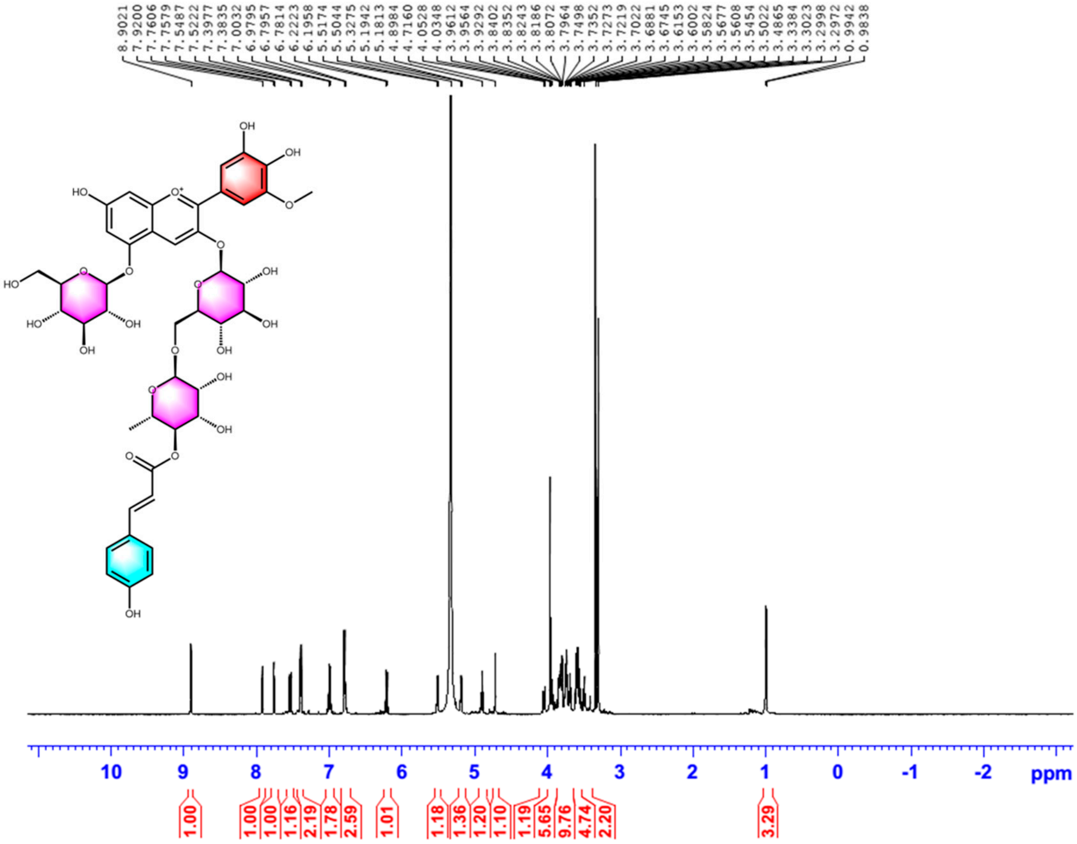

Figure S5. ^{1}H NMR and structure of petanin

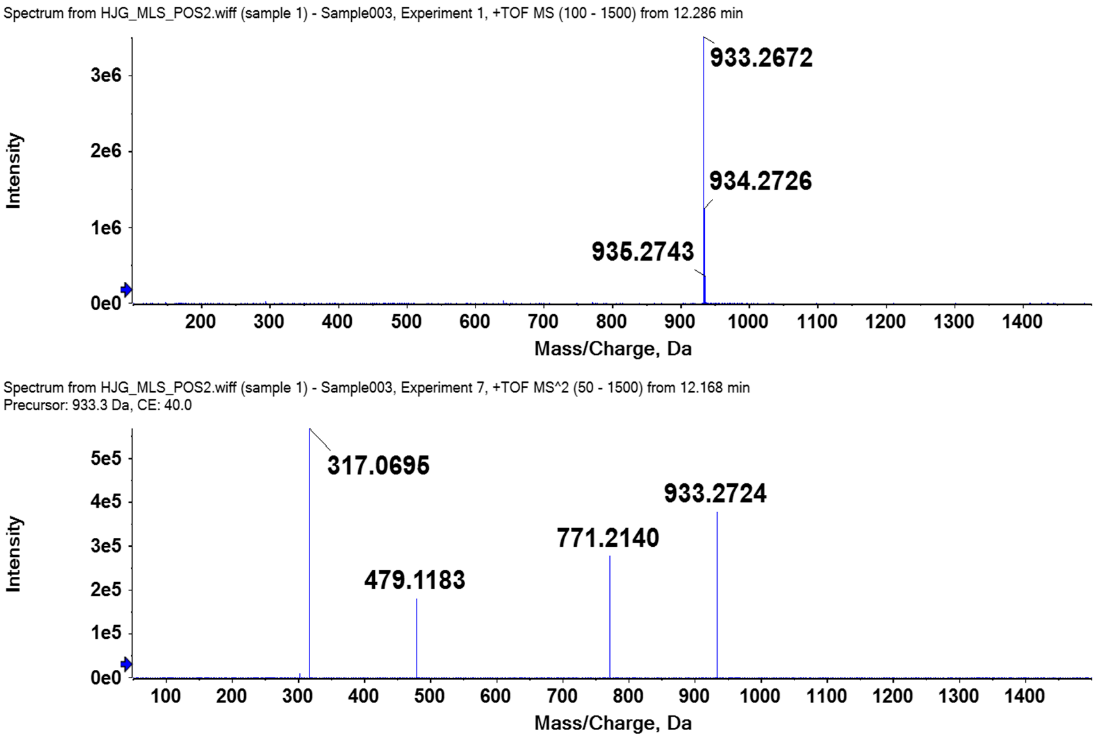

Figure S6. Mass spectrum of petanin from liquid chromatography-mass spectrometry

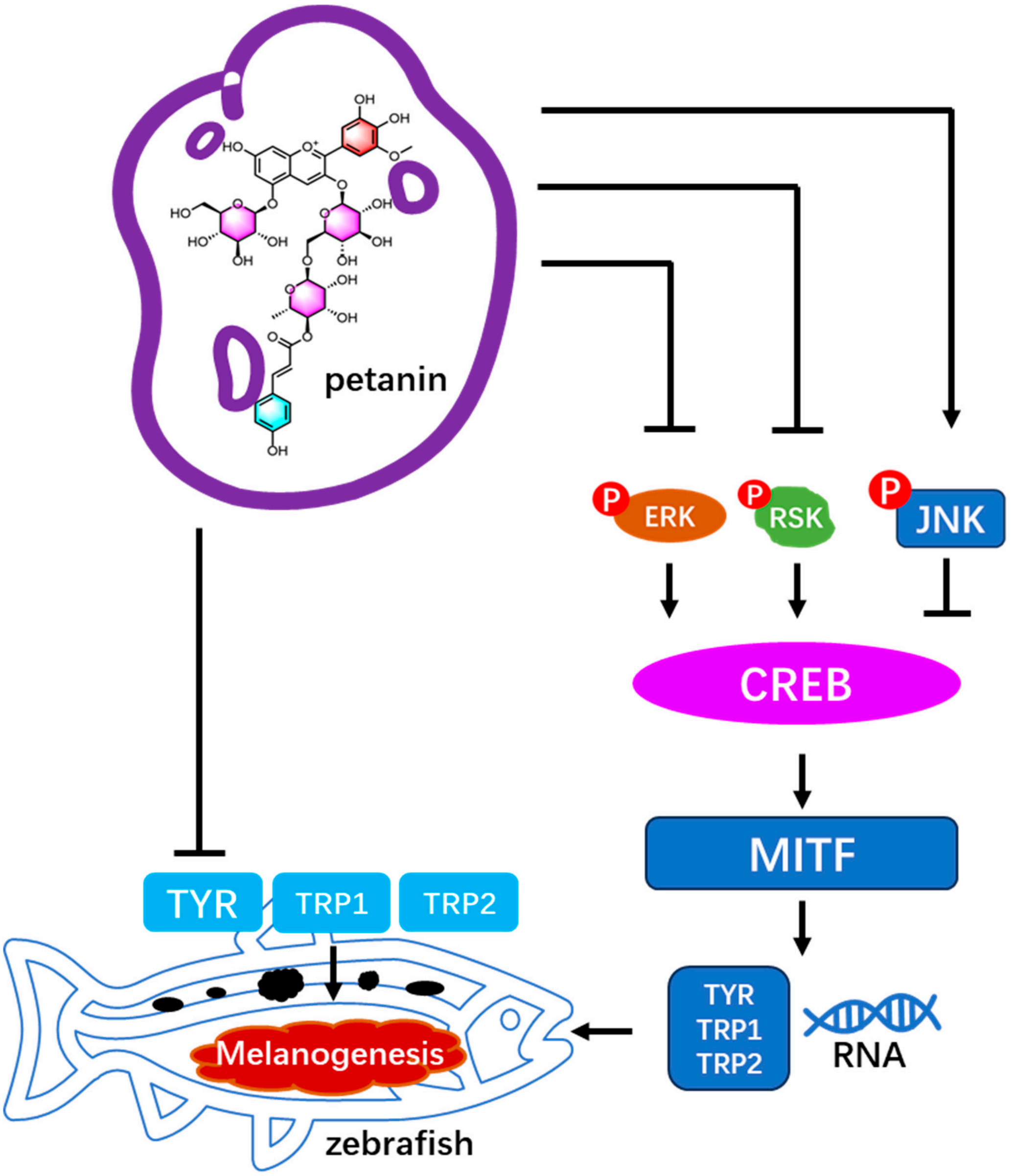

Figure S7. Effect of petanin on ERK/CREB/MITF signaling pathway

Table S1. Molecules docking of Petanin binding energy and hydrogen bonds with key signaling pathway protein

Compound	Protein	PDB ID	Amino acid	Distance（Å）	Affinity (kcal/mol)
Petanin	ERK	5KE0	ARG189	2.1/2.2	-9.8
			LYS149	2.0	
			GLU31	2.5	
			ALA33	2.1	
			TYR34	2.3/3.0	
			ARG65	3.0	
			ASP165	2.1	
	p38	3ZS5	ASP168	2.7	-9.4
			HIS107	2.5	
			MET109	3.0	
			TYR35	2.9	
			HIS64	3.2/3.0	
			THR68	3.0	
			ARG67	2.9/3.0	
	PKA	1CX4	TYR347	2.7/3.2	-8.4
			GLN346	3.2/2.8	
			GLY345	2.8	
			ARG344	3.0	
			ARG341	2.2	
			TYR397	3.3	
	JNK	3PZE	SER179	3.0	-10
			ARRG69	2.8/3.3	
			GLN37	3.2	
			GLY38	2.8	
			SER34	2.8/2.0/3.0/3/5	
			ILE32	2.0/3.0/2.8	
			MET111	2.8	

Table S2. Primers for mRNA associated with melanogenesis in zebrafish

Gene name	Primer
gadph	F: ACAGCAACACAGAAGACCGT
	R: ATACCAGCACCAGCGTCAAA
α-MSH (pomca)	F: GCCCCTGAACAGATAGAGCC
	R: CTTGATGGGTCTGCGTTTGC
mclr	F: TCATCTTGGTGGTGTGGCTTGC
	R: AAGAACGGTCCCCAGCAGAGG
creb	F: GCCATGTTCACTTACCATTGAG
	R: TTGTAAGAGGCATGTGAGATGG
mitf	F: TGTACAGCAATCATGCTCTTCC
	R: GTCCCCAGCTCCTTAATTCTGTC
trp1α	F: CTCATCATCGTCGCCATC
	R: GAACCTCCTGAAGAACACA
trp2	F: TGGACAGTAAACCCTGGGGA
	R: CCGGCAAAGTTTCCAAAGCA
tyr	F: GATCCAGGTCAGCGGTTTGT
	R: ACCGATGCGATTATTCCTGCT
MAPK1（ERK-2）	F: TCCACTCCGCAAACGTTCTT
	R: CATACTCCGTCAGGAAGCCG
rsk	F: CAGCATACTCCAGTCCCACC
	R: TACCGCAGCAGGATCTCAAC
p38α	F: TGGACATTTGGTCAGTGGGC
	R: CTGATGTAAGTACGAGCCTCATGG
MAPK8（JNK1）	F: TCGCTAGTTGGCATCGTTTATG
	R: CGGAGGTTCGAAGACGATCA
